# Pulmonary amyloidosis: a case report

**DOI:** 10.1093/omcr/omaf007

**Published:** 2025-03-28

**Authors:** Fenna Ahsino, Mouad Al Moudni, Jamal Eddine Bourkadi, Karima Marc

**Affiliations:** Department of Pulmonology, Moulay Youssef Hospital, Ibn Sina University Hospital, Rabat, Morocco; Department of Pulmonology, Moulay Youssef Hospital, Ibn Sina University Hospital, Rabat, Morocco; Department of Pulmonology, Moulay Youssef Hospital, Ibn Sina University Hospital, Rabat, Morocco; Department of Pulmonology, Moulay Youssef Hospital, Ibn Sina University Hospital, Rabat, Morocco

**Keywords:** amyloidosis, lung, nodule, dyspnea, biopsy

## Abstract

Amyloidosis is a rare disease in which abnormally folded proteins form accumulations called amyloid fibrils that accumulate in different tissues and organs, sometimes leading to organ dysfunction, organ failure and death. Rarely, pulmonary involvement can take one of three forms: nodular, diffuse alveolar-septal, or tracheobronchial. We report the case of a 60-year-old woman previously treated for confirmed pleural tuberculosis. She was admitted with progressive dyspnea for three months and a chest CT scan revealed a pulmonary nodule in the middle lobe. She underwent CT-guided biopsy and diagnosis of pulmonary amyloidosis was retained by histology. Follow up has been unremarkable.

## Introduction

Amyloidosis is a heterogeneous group of diseases, which results from extracellularly accumulation of improperly folded proteins, causing damage and dysfunction of organ [1]. The process of amyloidosis usually occurs in several stages: production of an abnormal protein because of a genetic mutation or excess in the body. Then, the protein does not fold properly, adopting a shape that allows it to clump together with other similar proteins and therefore the formation of fibrils where misfolded proteins assemble into long, insoluble fibers called amyloid fibrils which will accumulate in organs, disrupting their normal function and leading to diseases such as amyloidosis. Amyloidosis is divided according to the International Society of Amyloidosis (ISA) into systemic and localized forms. The most common types of amyloidosis are localized AL, systemic AL (primary) include immunoglobulin light-chain amyloidosis (AL) due to an underlying monoclonal B-lymphocyte/plasma cell disorder, systemic AA (secondary) due to chronic inflammatory diseases, systemic wild-type ATTR and systemic hereditary ATTR are age-related or familial, respectively, and involve the deposition of transthyretin protein [2, 3]. In 50% of the patients with amyloidosis, a respiratory involvement may develop. In patients with amyloidosis AL, the prevalence of pulmonary deposits ranges from 36% to 90% in histopathological studies [2]. The histological staining with Congo red is the gold standard to confirm the diagnosis of amyloidosis. Under a polarized light microscope, the amyloid deposits show an apple-green birefringence. Three manifestations of pulmonary amyloidosis are possible: nodular, diffuse alveolar-septal, or tracheobronchial [4]. The clinical symptoms are nonspecific and vary depending on localization. They can vary from dyspnea, cough to bronchial obstructions signs, which can mimic other pulmonary diseases [1]. Treatment with high-dose chemotherapy or autologous stem cell transplantation is indicated in systemic forms. While in localized form, a symptomatic treatment may remain [1]. We present a case of a 60-year-old patient diagnosed with nodular pulmonary amyloidosis. This aspect most often brings to mind at first glance other diagnoses such as cancers or bronchopulmonary infections. The differential diagnostic approach and management requires the use of modern imaging techniques primarily positron emission tomography (PET) and histological analysis remains the key examination for definitive diagnosis.

## Case report

**Figure 1:**
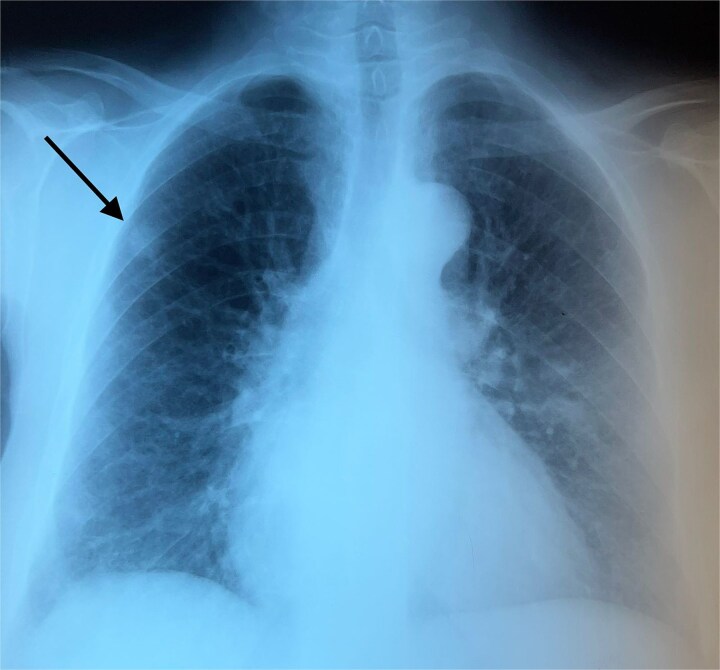
Chest X-ray showing a peripheral well-limited, rounded opacity in the right lung.

**Figure 2:**
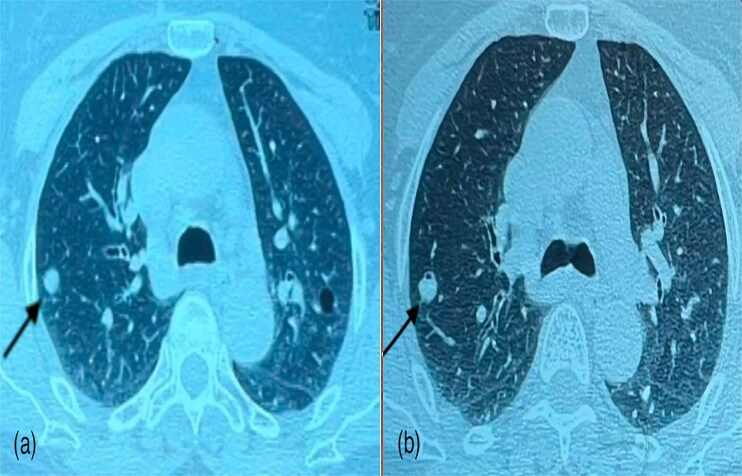
Parenchymal window images of CT scan schowing a pulmonary nodule in the middle lobe.

**Figure 3:**
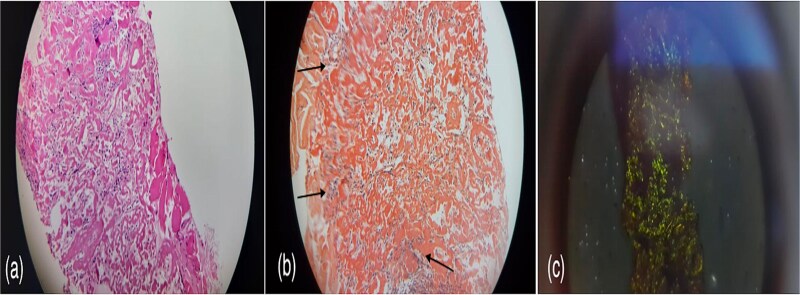
(a) Amorphous eosinophilic deposits, (b) brick red colored substance deposits on Congo-red staining and (c) apple-green birefringence in polarized light.

A 60-year-old woman with a history of confirmed pleural tuberculosis and without toxic habits. She presented with dyspnea with no other associated signs. Clinically, the patient was apyretic, with a performans status of 0, with no evidence of weight loss or abnormalities on physical examination. Biological tests was normal. The chest X-ray showed a peripheral well-limited, rounded opacity in the right lung (Fig. 1). Chest CT-scan revealed a pulmonary nodule in the middle lobe (Fig. 2). Bronchoscopy showed no endobronchial abnormality. As malignancy was suspected, she underwent CT-guided biopsy and subsequent pathology revealed amyloidosis with deposits stained with Congo red were positive and showed apple-green birefringence in polarized light (Fig. 3). The workup for visceral localization included a thorough clinical examination, revealing no signs of peripheral amyloidosis (macroglossia, peri-ocular lesion). Cardiac ultrasound was normal. Proteinuria was negative, so renal biopsy was not considered. Blood count was normal, with no abnormalities in circulating lymphocytes. The patient is currently under disease surveillance.

## Discussion

The amyloidosis is caused by the aberrant fibrillar protein deposition that causes the afflicted organ to gradually malfunction and maybe fail. They could be fatal or asymptomatic, inherited or acquired, localized or systemic [5].

Pulmonary involvement is rare. The Mayo Clinic and the Cordier *et al*. studies are the two most important. The American study described 55 patients, 35 with primary systemic amyloidosis (AL). Among the localized forms, 4 of tracheobronchial and 7 of nodular parenchymal involvement were reported. The French study described 21 patients, including 5 with tracheobronchial amyloidosis, 2 with parenchymal nodular amyloidosis and 15 with interstitial amyloidosis [5].

Whether systemic or localized, AL amyloid frequently affects the lungs [4, 6]. Bronchoscopy with biopsy is the main diagnostic tool for pulmonary amyloidosis [7]. In our case, bronchoscopy showed no endobronchial abnormality and as malignancy was suspected, our patient underwent CT-guided biopsy and subsequent pathology revealed amyloidosis. Immunohistochemical staining is used to identify the different subtypes. Under polarized light, amyloid exhibits apple-green birefringence when stained with Congo red [4, 6]. Amyloid can also be identified via thioflavine-T fluorescence staining and crystal violet staining [8]. Once a diagnosis has been made, chest CT imaging can be used to track the disease’s course and assess its extent.

Three forms of lung involvement are described: nodular, diffuse alveolar-septal, and tracheobronchial [4, 6].

Chest radiography often reveals nodular pulmonary amyloidosis incidentally. It is typically localized but can be bilateral and manifests as variable-sized peripheral subpleural localizations, unlike the malignant nodule where we find a spiculated nodule with irregular contours [4]. It is characterized by one or more nodular amyloid depsits in the lung. Immunoglobulin light chain (AL) or mixed immunoglobulin light and heavy chain (AL/HA) amyloidosis is usually represented by this form [9]. Conservative excision is typically used to treat nodular amyloidosis with a good long-term prognosis [4]. As in the case of our patient, whose chest scan revealed a pulmonary nodule in the middle lobe and whose histological study of the CT-guided biopsy revealed amyloidosis. We insist that in front of this scan aspect we must not miss a cancer or a bronchopulmonary infection.

Amyloid deposits in the alveolar septa and vessel walls are a characteristic of diffuse parenchymal amyloidosis, also known as diffuse alveolar-septal amyloidosis. It is typically a form of systemic amyloidosis. Reticular opacities, micronodules, interlobular septal thickening, and, less commonly, ground-glass opacification, bronchiectasias, and honeycombs can all be seen on a thoracic CT scan. Sometimes mediastinal lymphadenopathy may accompany it [5]. Its clinical manifestation is more severe. Patients present with dyspnea (not explained by heart involvement) and an infiltrative imaging pattern. Its caused by deposits affecting gas exchange in the interstitium. Vascular deposits are common and can cause pulmonary hypertension, however they are rarely clinically significant [4]. Treatment for this kind of amyloidosis is determined by the systemic amyloidosis that underlies it. Current strategies for treating AL amyloidosis are based on chemotherapy schemes created for multiple myeloma, which quickly and significantly lower the amount of circulating free light chain [10].

Multifocal submucosal plaques are the most common presentation of tracheobronchial amyloidosis. Though colocalisation of tracheal and laryngeal amyloidosis has been reported, the pulmonary parenchyma is normally unaffected [11]. Patients with tracheobronchial amyloidosis have a mean age of 50–60 years and no discernible sex predilection. The stenosis caused by amyloid deposits in the trachea and large bronchi frequently causes symptoms, such as cough, dyspnea, and hemoptysis [4]. Irregular whitish deposits, most frequently diffuse, are typically seen on bronchoscopy. Following a biopsy, the delicate lesions may bleed. Since there is no effective therapy for tracheobronchial amyloidosis, its management is primarily based on symptoms. Respiratory insufficiency and potentially fatal infections can result from tracheobronchial involvement.

Prognosis is good in Cordier’s study, with 3 out of 4 patients surviving beyond 8 years. In the Mayo Clinic study showed a poor prognosis, with 75% of patients dying within 79 months [5]. In our patient, she has a good prognosis and she is currently under disease surveillance.

## Conclusion

From asymptomatic incidental discovery to potentially lethal disease; pulmonary amyloidosis is rare. Nodular, diffuse alveolar-septal, and tracheobronchial lung involvement are the three forms of lung involvement that are characterized. We discussed a rare case of pulmonary involvement of amyloidosis in its nodular form, diagnosed by CT-guided biopsy, in a 60-year-old woman. Histological confirmation is crucial in ruling out malignancy, as this case demonstrates. The main differential diagnoses for nodular form are neoplasms, granulomatous lung diseases, mucosa-associated lymphoid tissue (MALT) lymphomas and connective tissue diseases. When faced with a pulmonary nodule in a patient in good general condition who has no risk factors for bronchopulomarary cancer, it is also necessary to think about pulmonary amyloidosis which will be confirmed by histology.
